# Tobacco smoking as an endocrine disrupting chemical: An assessment through biological monitoring

**DOI:** 10.18332/tid/205064

**Published:** 2025-07-29

**Authors:** Ho-Sun Lee, Hyun-Kyung Na, Seong-Sil Chang, Soo-Young Kim, Chang Seong Kim, Min Ju Kim, Mihi Yang

**Affiliations:** 1 National Forensic Service, Daegu, Republic of Korea; 2 College of Pharmacy, Sookmyung Women’s University, Seoul, Republic of Korea; 3 Department of Occupational and Environmental Medicine, Changwon Hospital of Korean Workers' Compensation and Welfare Service, Changwon, Republic of Korea; 4 Department of Occupational and Environmental Medicine, Eulji University Hospital, Daejeon, Republic of Korea; 5 Korea Testing and Research Institute, Gwacheon-Si, Republic of Korea; 6 College of Dentistry, Chosun University, Gwangju, Republic of Korea

**Keywords:** tobacco smoking, biological monitoring, adolescent, genetic polymorphism, mtDNA alteration

## Abstract

**INTRODUCTION:**

Tobacco smoke is a mixture of endocrine disrupting chemicals (EDCs), which may accelerate biological ageing.

**METHODS:**

Within this cross-sectional study we recruited adult and adolescent subjects (2013–2014) and performed biological monitoring to clarify health end points of tobacco smoking between adolescents and adults (n=620) with exposure biomarkers, i.e. CO, urinary cotinine, t,t-muconic acid (TTMA), malondialdehyde (MDA), and obtained information of behavioral factors and tobacco addiction status in South Korea. We also analyzed the 96 SNPs for metabolism, addiction, and expression differences and compared mtDNA abnormalities in buccal and blood cells.

**RESULTS:**

There was an association between tobacco smoking and oxidative stress with urinary cotinine and MDA levels. Youth smokers showed lower frequency in some of mtDNA alteration, SNPs for consistent bases between buccal and blood cells, than youth non-smokers or adult smokers. Among the SNPs, the polymorphisms on SULT1A1, DRD2, and ADH1B were related to multiple of the above exposure biomarkers. Interestingly, urinary MDA or TTMA in youth were similar to those in adults (MDA, 2.7 ± 1.5 vs 2.4 ± 1.3 μM; TTMA, 74.1 ± 129.9 vs 98.8 ± 126.1 μg/L), although urinary cotinine levels were approximately four-fold lower in youth than adults (0.1 ± 0.4 vs 0.6 ± 0.9 mg/L; p<0.0001). Urinary MDA, an oxidative stress biomarker, were negatively associated with the growth rate among the adolescents.

**CONCLUSIONS:**

The present biological monitoring study assessed the impact of combustible cigarette smoking with various exposure, susceptibility and response biomarkers to clarify how tobacco smoking differently affects adolescents and adults in South Korea.

## INTRODUCTION

Endocrine disrupting chemicals (EDCs) are a heterogeneous group of exogenous compounds that can restrict with several facets of endogenous hormones and accelerate aging, immune, metabolic or neurobehavioral disorders to threaten quality of life^1^. Persistent exposure to EDCs can disrupt homeostasis in the body and creates oxidative stress that can lead to aging and chronic inflammation^2^. These characteristics were also found to be significant in the observation of telomere length, which is a measure of aging^3^. As aging is a complex process influenced by genetic, environmental, and lifestyle factors, changes in endogenous hormone levels are part of this intricate interplay^4^.

Tobacco smoke is an environmental mixture with over 7000 chemicals^5^ and is the main indoor source of polycyclic aromatic hydrocarbons (PAHs)^4^. It may obstruct and interfere in the function of endocrine system and has been entitled as EDCs^6^. PAHs are known to produce bulky DNA lesions that are considered to play a major role in smoke-induced mutagenesis and carcinogenesis^7^. In addition, the mutagenic effects of tobacco smoking as EDCs have been previously associated with male fertility^8^ and with intellectual disability-associated genes with approximately 1.7 and 0.2 million *de novo* mutations on the autosomes and the X chromosome, respectively^9^.

With regard to the molecular toxic mechanisms of tobacco, oxidative stress or inflammasome activation as well as a chronic activation of aryl hydrocarbon receptor signaling can contribute to premature aging and the development of neoplasms by affecting metabolism, extracellular matrix remodeling, inflammation, pigmentation, DNA repair, and apoptosis^10^. In addition, exposure timing and early life exposure to tobacco smoking in children or youth can be manifested into adulthood via cellular memory, and lead to epigenetic changes^11^.

For exposure biomarkers of tobacco smoking, parent chemicals in tobacco smoke or their specific metabolites have been used^12^. The most representative biomarkers are cotinine, a metabolite of nicotine, and t,t-muconic acid (TTMA), a metabolite of benzene, which are quite attributable to their specificity and sensitivity among the various urinary metabolites of tobacco^13,14^. In addition, altered biological responses to tobacco smoke exposure have potential as early diagnostic biomarkers. For example, alteration in genomic and mitochondrial (mt) DNA or oxidative stress parameters, such as malondialdehyde (MDA)^15^, can be considered as popular biomarkers as responsive to tobacco smoking.

Therefore, we performed a molecular epidemiological approach with in-depth biological monitoring of the impact of combustible cigarette smoking with various exposure, susceptibility and response biomarkers to clarify how tobacco smoking differently affects adolescents and adults in South Korea, where the current smoking rate of adolescents was 4.5% in 2022 and for adults was 17.7% in 2022 ^16^.

## METHODS

### Subjects and sampling

We performed a cross-sectional study, to clarify health end points of tobacco smoking between adolescents and adults (recruited, n=679; missing, n=59; bio-monitored, n=620) and recruited the adults and adolescents (2013–2014) from four sites (Table 1). The adults visited Eulji University Hospital in Daejeon, South Korea, for regular examination, and adolescents from high schools around Geumsan-gun near Daejeon. All subjects provided written informed consent and completed extensive questionnaires including medical and smoking history, dietary patterns, alcohol drinking, environment of residence, and smoking behaviors, such as smoking cessation, self-reported cigarettes per day (CPD), duration of smoking, and smoking initiation. In addition, the Fagerström test of nicotine dependence (FTND) was used to assess nicotine dependence^17^. We defined non-smokers, who did not smoke for the last one year. Thus, ex-smokers were included in the non-smoker group. None of these subjects had any history of pulmonary, cardiovascular, endocrine, or gastrointestinal disorders. In addition, we measured carbon monoxide (CO) during exhalation with Micro CO Monitor (On-site Lab, Seoul, Korea).

**Table 1:** Descriptive characteristics of the subjects based on recruitment site, South Korea

*Sites*	*Age (years)* *Mean ± SD*	*Non-smoker* *n (%)*		*Smoker* *(n %)*		*Row total* *n (%)*
		*Male*	*Female*	*Male*	*Female*	
**Regular examination**	42.23 ± 5.47	78 (37.3)	87 (41.6)	44 (21.1)	0 (0)	209 (33.7)
**Occupational examination**	38.31 ± 7.28	73 (41.7)	13 (7.4)	89 (50.9)	0 (0)	175 (28.2)
**Smoking cessation clinic**	42.03 ± 7.30	0 (0)	0 (0)	73 (100)	0 (0)	73 (11.8)
**High school**	16.35 ± 0.53	50 (30.7)	87 (53.4)	26 (16.0)	0 (0)	163 (26.3)
**Column total**		201 (32.4)	187 (30.2)	232 (37.4)	0 (0)	620 (100)

Peripheral blood samples (10 mL) were collected into evacuated tubes containing sodium heparin as an anticoagulant (BD Vacutainer, Franklin Lakes, NJ, USA). In addition, spot urine specimens, the first voids of urine (40 mL) before breakfast, were collected into 50 mL of conical tubes. Both urine and blood samples were stored at -20^o^C until analyses. All study protocols for this study were approved by the Institutional Review Board of Eulji University Hospital (ID:201308004, approved date, Sep., 10, 2013).

To compare mtDNA alteration in buccal cells to that in blood cells, we also collected buccal cells from the subjects with sterile cotton swabs, following our previous method^18^.

### Analyses of urinary cotinine

We analyzed urinary cotinine, a metabolite of nicotine as an exposure biomarker for tobacco smoking, by our previous ion-pair HPLC/UVD method^18^ with minor modifications. In brief, 900 μL of each urine sample were mixed with 100 μL of 80 μM 2-phenylimidazole as an internal standard and 330 μL of 3 M NaOH. The mixture was twice extracted with 3 mL of CH_2_Cl_2_ each time. After evaporating CH_2_Cl_2_ -extract, we dissolved the residue in 1 mL of water and injected 20 μL of its supernatant fraction to HPLC. The HPLC system consisted of dual Younglin SP930D pumps (Younglin, Seoul, Korea), a MIDAS COOL autosampler (Spark Holland, Emme, The Netherlands), an SPD-10A UV-VIS detector (Shimadzu, Kyoto, Japan), and a TSK gel ODS-80™ column (5 μm, 4.6 mm × 150 mm, Toyo Soda Co., Tokyo, Japan). Analyses were carried out with the following gradient mode: mobile phase A, a mixture of acetonitrile/water (15/85) containing 20 mM KH_2_PO_4_ and 3 mM sodium 1-octanesulfonate (pH 4.5); B, methanol; Flow rate, 0.7 mL/min; 0–20 min, ratio of A to B = 100:0; 20–25 min, ratio of A to B = 100:0 to 50:50; 25–30 min, ratio of A to B = 50:50; 30–35 min, ratio of A to B = 50:50 to 100:0; and 35–45 min, ratio of A to B = 100:0. The column was kept at 50^o^C and the absorbance was observed at wavelength of 254 nm^18^.

### Analyses of urinary MDA

We quantified urinary MDA, as an oxidative biomarker, with adducts of 2-thiobarbituric acid (TBA, CAS number: 504-17-6) with HPLC/UVD^19^. TBA-MDA adducts were detected at 532 nm with isocratic mode. The mobile phase was a mixture of 50 mM potassium phosphate buffer (pH 6.8) and methanol (58:42, v/v). Flow rate was set at 0.6 mL/min.

### Analyses of urinary TTMA

We analyzed urinary TTMA, a metabolite of benzene as an exposure biomarker for tobacco smoking, with UPLC-MS/MS, using the method previously described by Gagne et al.^20^ with a minor modification. Briefly, TTMA standard and deuterated internal standard, d4-TTMA, were obtained from Sigma-Aldrich and CDN Isotopes Inc. (Pointe-Claire, Quebec, Canada), respectively. Standard TTMA solutions (0.025–2.5 ng/mL) were prepared in 50% methanol. In short, 950 μL of 0.1 % formic acid containing 2.38 μg/mL of d4-TTMA was mixed with 50 μL of TTMA standards or urine samples. After centrifugation at 13000 rpm for 10 min, 5 μL of each supernatant was analyzed with UPLC-MS/MS. The UPLC-MS/MS system consisted of a Waters Acquity UPLC coupled with a Waters Xevo TQ triple quadrupole mass spectrometer (Beverly, MA), and an Acquity UPLC BEH C18 (1.7 μm, 2.1 mm × 50 mm, Waters). Mobile phases were composed of 0.1% formic acid in methanol (eluant A) and in water (eluant B). UPLC separation was achieved with a gradient from 10 to 95% of eluant A for 1.25 min. Eluant A composition was then held constant for 0.5 min followed by a 0.5 min equilibration period at 10% of eluant A. The flow rate was set at 0.5 mL/min and the column temperature was kept at 50^o^C. The Xevo TQ was operated in negative mode. The capillary voltage was set at 2.8 kV. The source temperature was at 150^o^C. The desolvation temperature was at 500^o^C. Desolvation flow rate was at 900 L/h and collision gas flow rate was at 0.15 mL/min. Data were acquired in multiple reaction monitoring (MRM) mode.

Urinary cotinine, MDA, and TTMA were adjusted for creatinine, measured with ion-pair HPLC/UVD method^18^. Limits of quantification (LOQ) for cotinine, MDA and TTMA were 0.015 mg/L, 0.06 μM, and 0.1 μg/L, respectively, and limits of detection (LOD) for them were approx. 1/3 of the above LOQs.

### Targeted genotyping

Genomic DNA of peripheral blood was isolated with a QIAamp DNA Blood Mini kit (Qiagen, Hilden, Germany) according to the manufacturer’s instructions. We measured the purity and concentration of the isolated genomic DNA (gDNA) using a NanoDrop® ND-1000 spectrophotometer (NanoDrop Technologies, Wilmington, DE). For the genotyping, we used gDNA samples with a 260:280 ratio of 1.5 or higher and concentrations of 60 ng/μL or higher.

We selected the 96 target SNPs, based on the SNPs of tobacco smoking-responsive genes, of which expression levels were altered by tobacco smoking in our previous microarray study18, i.e. ACTG1, DEFA4, VAV3, FCGR3A, etc.; as well as the SNPs related to metabolism, e.g. CYP2A6, CYP1B1, CYP2E1, and NQO1; addiction^21^, e.g. CHRNA5/A3/B4, 5-HTTLPR, and DRD2 ^22^; risks for lung cancer, e.g. CYP1A1, GSTP1, and MPO1^22,23^; DNA repair, e.g. ERCC1, MGMT, XRCC1, etc.^24^; and epigenetic modulation, e.g. HDAC1 and MTHFR^25^ of tobacco smoking (Supplementary file Table 1). For genotyping assays, the 96.96 Dynamic Array™ integrated fluidic circuits (Fluidigm Corp., South San Francisco, CA) was used. Prior to genotyping, specific target amplification (STA) was performed for each gDNA to enrich targeted SNP sequences. Briefly, 70 ng of gDNA was mixed with 50 nM of STA primer mixture, 50 nM of locus specific primer mixture, and 2.5 μL of Qiagen 2X Multiplex PCR Master Mix (Qiagen) in a final volume of 5μL. PCR reactions were performed on an Arktik Thermal Cycler (Thermo Scientific, Rockford, IL) with the following cycling conditions: 10 min at 95°C, followed by 14 cycles of a 2-step amplification profile of 15 s at 95°C and 4 min at 60°C. STA products were 100-fold diluted in DNA suspension buffer and 2.5 μL of each product was combined with 3.0 μL of 2X Maxima® Probe/ROX qPCR Master Mix (Fermentas, St. Leon-Rot, Germany), 0.3 μL of SNPtype 20 X Sample Loading Reagent (Fluidigm), 0.1 μL of SNPtype Reagent (Fluidigm), and 0.1 μL of nuclease-free water. In parallel, 1 μL of each SNPtype assay was mixed with 2.5 μL of 2 X Assay Loading Reagent (Fluidigm) and 1.5 μL of nuclease-free water and loaded into the Fluidigm 96.96 Dynamic Genotyping Arrays. PCR and image processing were carried out on an EP1 system (Fluidigm). We analyzed data with an automated genotype calling algorithm using Fluidigm SNP Genotyping Software (v3.1.1).

### Analyses of mtDNA alteration between blood and buccal cells

After extraction of DNAs from buccal cells and blood with DNeasy blood & tissue kit (Qiagen), we prepared a 96 well plate (Bioline, London, UK) to load 50 μL of DNA samples (5 ng/μL =250 ng). From the master plates, 5 ng of DNA was used for a conventional PCR to amplify HV2 region. Total reaction volume was 20 μL, containing 5 ng DNA and 75 μM of forward and backward primers, F015 5’-CAC CCT ATT AAC CAC TCA CG-3’ and R569 5’-GGT GTC TTT GGG GTT TGG TTG-3’, respectively. PCR conditions for amplification were performed on Primus 96 plus (MWG_Biotech, Huntsville, AL): initial denaturation at 96°C for 5 min; 35 cycles of denaturation at 96°C for 30 s; annealing at 56°C for 30 s; elongation at 72°C for 1 min, with MyGenieTM 96 Gradient Thermal Block (Bioneer, Daejeon, Korea). For sequencing target mtDNA, we used BigDye Terminator v3.1 sequencing kit and ABI3730XL (Applied Biosystems, Waltham, MA).

For data analyses, we used DNASTAR Lasergene SeqMan Pro version 7.1.0. Among the reference sequence of the mtDNA HV2 region (015-560 bp), NCBI (ref|NC_012920.1) corresponding location from 100 bp to 322 bp of mtDNA HV2 region was analyzed for detecting discrepancies between blood and oral DNA on (a/g or t/c) polymorphism at the position 263 bp and polyC region [303–314 in reference C7(T)C5].

### Functional enrichment

We used protein-protein interaction analyses with STRING 12.0 and functional annotation in GO Biological Process 2023 to investigate that the epigenetically modified genes played a systemic role in various pathways. We ranked enriched terms in our results using Enrichr, which includes gene set libraries.

### Statistical analysis

We removed imputed SNPs with <0.05 genotype information content, low call rates with <0.90, and minor allele frequency (MAF) with <0.05. The Shapiro-Wilk W test was used to test distributional normality for levels of exposure biomarkers (i.e. urinary cotinine, TTMA and MDA). Regression analyses were performed among continuous levels of biomarkers. ANOVA or Wilcoxon rank sum test was used to analyze differences in biomarker levels by smoking and various mtDNA alteration by smoking and gender. Kruskal-Wallis test was used to analyze associations between genotypes and levels of exposure biomarkers. All test were two-tailed and p<0.05 was considered to be statistically significant. All statistical analyses were conducted with JMP package v. 4.0.2 (SAS Institute, Cary, NC).

## RESULTS

### Characteristics of subjects

The ratio of non-smokers and smokers were similar, approx. 32–37% among men. Thirty-four percent of the recruited male youths smoked combustible cigarettes. Adult smokers smoked 18.5 ± 9.7 pack-years and started tobacco smoking at the age of 21.8 ± 6.2 years. There were no significant differences in age and body mass index (BMI) due to smoking in the adults. However, education years and alcohol drinking were negatively (p<0.05) and positively (p<0.001) related to smoking, respectively.

For youths, they smoked on average 2.2 ± 1.2 pack-years and started tobacco smoking at the age of 13.8 ± 2.3 years. The nicotine dependence scores by FTND for adults and youth were 3.2 ± 2.3 and 0.7 ± 0.9, respectively. The nicotine dependence status of most of the subjects was relatively low; however, it was higher in adults than adolescents.

### Exposure levels of tobacco smoking

The CO levels of exhaled gas were approximately five-fold higher in smokers than in non-smokers (13.4 ± 8.3 ppm vs 2.8 ± 2.6 ppm, p<0.001). For urinary exposure biomarkers, the ranges of urinary cotinine, TTMA and MDA were 0.015–4.4 mg/L (median: 0.015 mg/L), 0.1–1324.6 μg/L (median: 37.7 μg/L) and 0.06–13.3 μM (median: 2.5 μM). When we compared the exposure levels by smoking, the smokers showed significantly higher in most of the biomarkers than non-smokers (Table 2), although the association somewhat decreased after creatinine adjustment.

**Table 2:** Differences in exposure biomarker levels between smokers and non-smokers, South Korea

*Biomarkers*	*Non-smoker* *Mean ± SD*	*Smoker* *Mean ± SD*	p*
CO (ppm)	2.83 ± 2.56	13.37 ± 8.28	<0.001
Urinary cotinine (mg/L)	0.05 ± 0.21	0.83 ± 1.00	<0.001
Urinary cotinine (mg/g creatinine)	0.04 ± 0.16	0.76 ± 1.06	<0.001
Urinary TTMA (μg/L)	42.44 ± 64.30	123.28 ± 203.33	<0.001
Urinary TTMA (μg/g creatinine)	43.30 ± 68.24	101.90 ± 183.83	0.02
Urinary MDA (μM)	2.38 ± 1.16	2.76 ± 1.46	0.04
Urinary MDA (μM/g creatinine)	1.92 ± 0.85	2.00 ± 1.21	0.86

* Wilcoxon rank-sum test.

**Figure 1:**
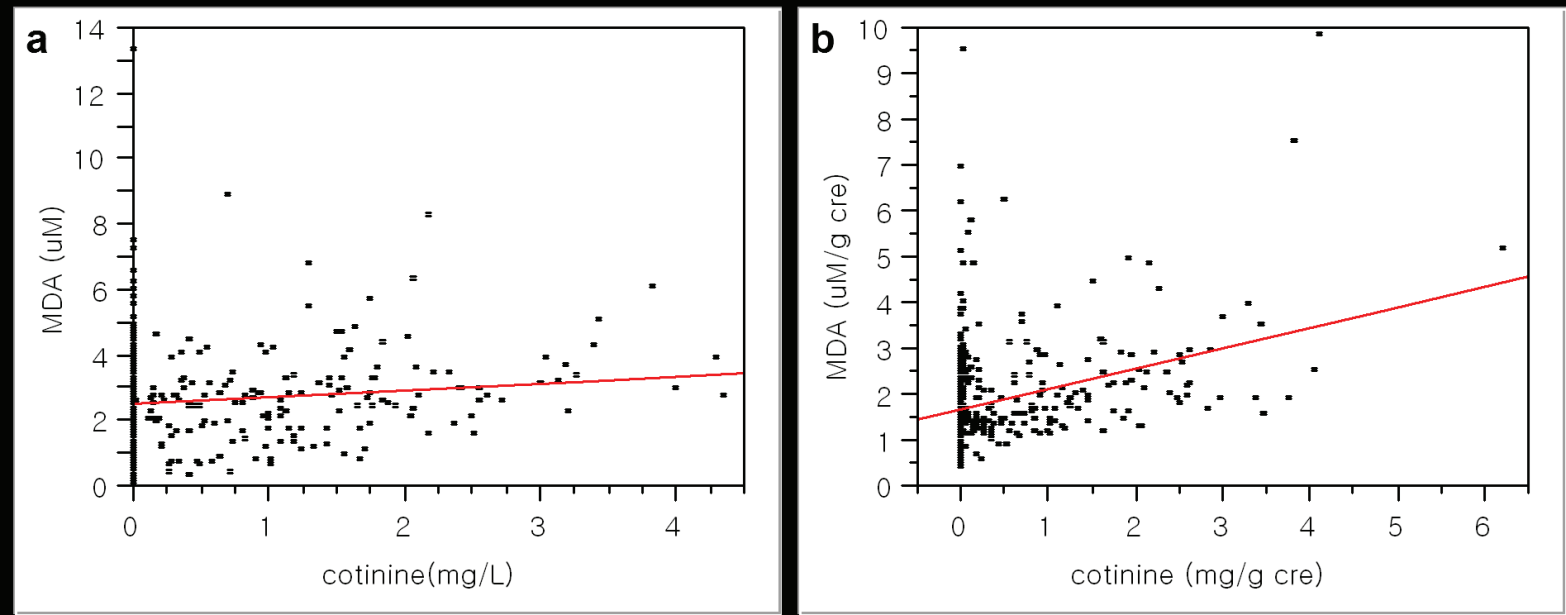
Association between tobacco smoking and oxidative stress as urinary cotinine and MDA levels: a) without creation modification, p<0.01, slope (estimate)=0.21, r^2^=0.01 by regression analysis; b) with creation modification, p<0.01, slope (estimate)=0.45, r^2^=0.12 by regression analysis (N=538)

**Figure 2:**
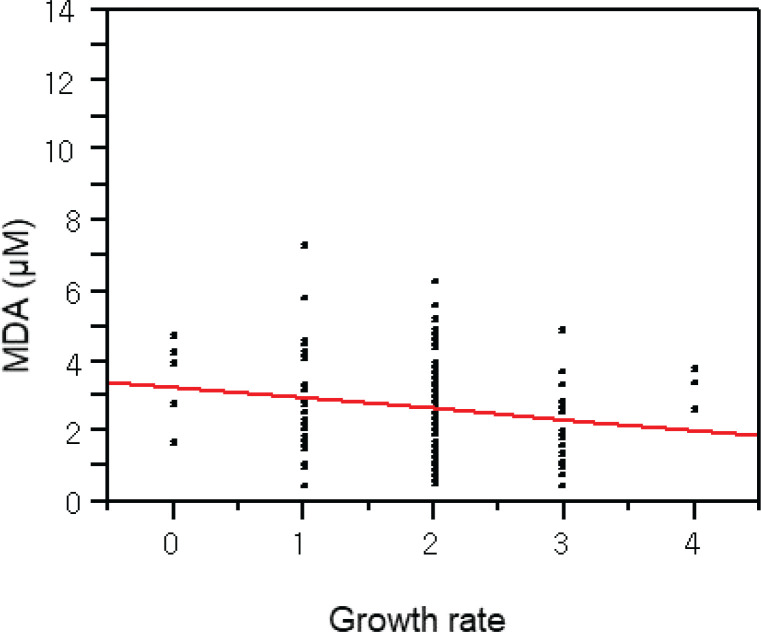
Negative association between oxidative stress and growth rate in adolescents: p=0.04, slope= -0.31, r^2^=0.18 by regression analysis (N=129); self-reported growth rate: 0, very slow; 1, slow; 2, normal; 3, fast; 4, very fast

In addition, there were strong positive associations among the exposure markers. For example, there was the association between tobacco smoking and oxidative stress with urinary cotinine and MDA (Figure 1). Interestingly, the self-reported growth rate in youth was negatively related to the MDA levels (Figure 2). Due to approximately six-fold higher packs per year of smoking in adults than youth, we expected quite big differences in these exposure by smoking. As results, urinary MDA or TTMA in youth were similar to those in adults (MDA, 2.7 ± 1.5 vs 2.4 ± 1.3 μM; TTMA, 74.1 ± 129.9 vs 98.7 ± 126.1 μg/L), although urinary cotinine levels were approximately four-fold lower in youth than adults (0.1 ± 0.4 vs 0.6 ± 0.9 mg/L; p<0.0001).

We also found positive associations between urinary cotinine and TTMA (r=0.38, p<0.01) and between urinary MDA and TTMA (r=0.25, p<0.01).

### mtDNA alteration by tobacco smoking

We found some differences in mtDNA alteration by smoking and gender (Supplementary file Table 2). In addition, we studied the differences in mtDNA alteration by smoking and age. As shown in Table 3, youth smokers showed somewhat high SNPs for inconsistent bases and significantly lower SNPs for consistent bases between buccal and blood cells than youth non-smokers or even adult smokers. Thus, youths showed vulnerability in mtDNA stability, compared to adults.

**Table 3:** Alteration of mtDNA by smoking and age

Item*	*Group*	*Mean*	*SD*	*P*	
Deletion of bases inconsistent between buccal and blood cells	Adult non-smoker	0.01	0.09	0.49	
	Adult smoker	0.00	0.00		
	Youth non-smoker	0.00	0.00		
	Youth smoker	0.00	0.00		
Deletion of bases consistent between buccal and blood cells	Adult non-smoker	0.09	0.29	0.53	
	Adult smoker	0.07	0.26		
	Youth non-smoker	0.13	0.33		
	Youth smoker	0.12	0.33		
Deletion of bases in blood cells	Adult non-smoker	0.00	0.06	0.75	
	Adult smoker	0.01	0.09		
	Youth non-smoker	0.00	0.00		
	Youth smoker	0.00	0.00		
Deletion of bases in oral (buccal) cells	Adult non-smoker	0.00	0.06	0.75	
	Adult smoker	0.00	0.00		
	Youth non-smoker	0.00	0.00		
	Youth smoker	0.00	0.00		
SNPs for inconsistent bases between buccal and blood cells	Adult non-smoker	0.35	10.01	0.49	
	Adult smoker	0.40	1.75		
	Youth non-smoker	0.49	1.38		
	Youth smoker	0.73	1.76		
SNPs for consistent bases between buccal and blood cells	Adult non-smoker	1.26	1.27	0.00	
	Adult smoker	1.12	1.06		0.00
	Youth non-smoker	1.23	1.56	0.00	
	Youth smoker	0.5	0.91		
SNPs in blood cells	Adult non-smoker	0.16	0.57	0.69	
	Adult smoker	0.16	0.68		
	Youth non-smoker	0.25	0.81		
	Youth smoker	0.27	0.7		
SNPs of bases in oral (buccal) cells	Adult non-smoker	0.19	0.79	0.71	
	Adult smoker	0.25	10.58		
	Youth non-smoker	0.22	00.88		
	Youth smoker	0.46	1.50		

Adult non-smoker, n=244. Adult smoker, n=134. Youth non-smoker, n=134. Youth smoker, n=26. Total, n=538 (some samples were discarded for statistical analyses, due to unclear mutation results). Data show mean of ‘mutation frequency/number of subjects’ with standard deviation (SD). Wilcoxon rank-sum and Kruskal-Wallis tests were used to compare two groups and over two groups, respectively.

* Deletion of bases or substitution of bases (SNP) between 100-322 bp of HV2 region.

### Genetic polymorphisms affecting exposure biomarkers

**Figure 3:**
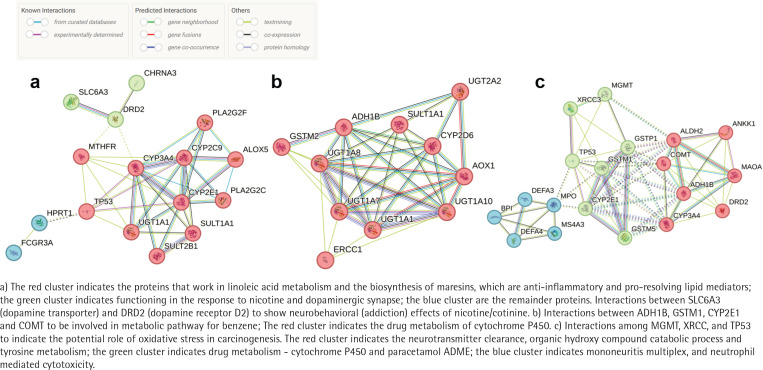
Protein-protein interactions by STRING 12.0 in SNPs with: a) urinary cotinine levels; b) t,tmuconic acid levels; and c) MDA levels

We could determine genotypes in the 15–86% of the gDNA samples for the 96 SNPs and found genetic polymorphisms affecting exposure biomarkers (Table 4). Six, seven, and ten genotypes among the 96 SNPs were associated with the levels of urinary cotinine, TTMA and MDA, respectively. The polymorphisms of some genes, such as SUL1A1 (rs9282861), ADH1B1 (rs1229984), and DRD2 (rs1800497), were even related to two different exposure biomarkers, i.e. cotinine and TTMA, TTMA and MD, or cotinine and MDA. Using these results, we performed pathway analyses to assess toxic mechanisms of tobacco smoking. As results, we can infer a neurobehavioral (addiction) mechanism from the interaction between SLC6A3 (dopamine transporter) and DRD2 (dopamine receptor D2) (Figure 3A). In addition, a group of metabolic enzymes for TTMA-associated polymorphisms on ADH1B, GSTM1, CYP2E1 and COMT can be involved in metabolic pathway for benzene (Figure 3B). Finally, three genes, MGMT, XRCC, and TP53, were associated with MDA to indicate the potential role of oxidative stress or aging in carcinogenesis (Figure 3C).

**Table 4:** Association between cotinine and associated SNPs

*Urinary cotinine*				
*Gene name (rs number)*	*Genotype*	*n*	*Cotinine (mg/L)*	p*
SULT1A1 (rs9282861)	AA	13	0.81 ± 0.20	**<0.01**
	AG	65	0.95 ± 0.09	
	GG	335	0.25 ± 0.04	
SLC6A3 (rs27072)	CC	39	0.27 ± 0.11	**<0.01**
	CT	28	0.02 ± 0.13	
	TT	156	0.48 ± 0.06	
MTHFR (rs1801133)	CC	154	0.58 ± 0.06	**<0.01**
	CT	276	0.27 ± 0.05	
	TT	107	0.39 ± 0.07	
ARTN (rs2853224)	AA	64	0.63 ± 0.09	**<0.01**
	AC	193	0.34 ± 0.05	
	CC	213	0.22 ± 0.05	
FCGR3A (rs396991)	GG	13	0.45 ± 0.21	**0.02**
	GT	209	0.27 ± 0.05	
	TT	295	0.45 ± 0.04	
DRD2 (rs1800497)	CC	223	0.44 ± 0.05	**0.01**
	CT	196	0.29 ± 0.05	
	TT	69	0.17 ± 0.09	
ALOX5 (rs7099684)	AA	7	0.86 ± 0.29	**0.01**
	AT	114	0.53 ± 0.07	
	TT	416	0.34 ± 0.04	
CHRNA3 (rs578776)	CC	14	0.23 ± 0.20	**0.04**
	CT	182	0.50 ± 0.06	
	TT	341	0.33 ± 0.04	
CYP2E1 (rs3813867)	CC	58	0.83 ± 0.10	**<0.01**
	CG	125	0.25 ± 0.07	
	GG	295	0.29 ± 0.04	
DEFA4 (rs2738102)	CC	85	0.66 ± 0.08	**<0.01**
	CT	241	0.32 ± 0.05	
	TT	206	0.36 ± 0.05	
HPRT1 (rs6634990)	GG	291	0.44 ± 0.04	**<0.01**
	GT	85	0.03 ± 0.08	
	TT	155	0.48 ± 0.06	
TP53 (rs1295105)	AA	277	0.44 ± 0.04	**0.04**
	AC	225	0.36 ± 0.05	
	CC	34	0.10 ± 0.13	
CYP3A4 (rs2242480)	CC	279	0.47 ± 0.05	**0.03**
	CT	144	0.42 ± 0.07	
	TT	57	0.16 ± 0.11	
***Urinary TTMA***				
***Gene name (rs number)***	***Genotype***	***n***	***TTMA (mg/L)***	***p***
SULT1A1 (rs9282861)	AA	13	203.46 ± 43.40	**<0.01**
	AG	65	165.60 ± 19.40	
	GG	335	64.29 ± 8.55	
ADH1B (rs1229984)	AA	67	81.09 ± 17.85	**<0.01**
	AG	111	59.11 ± 13.87	
	GG	26	267.63 ± 28.66	
CYP2D6 (rs3502862)	AA	92	65.54 ± 15.96	**0.02**
	AC	206	68.63 ± 10.67	
	CC	240	104.85 ± 9.88	
AOX1 (rs1759362)	CC	40	133.99 ± 17.65	**<0.01**
	CT	139	67.70 ± 9.47	
	TT	209	52.38 ± 7.72	
GSTM2 (rs638820)	CC	70	50.49 ± 18.25	**0.04**
	CT	184	74.41 ± 11.26	
	TT	283	97.79 ± 9.08	
IL17A (rs4711998)	AA	183	59.74 ± 9.35	**0.02**
	AG	99	62.96 ± 12.71	
	GG	132	98.82 ± 11.00	
ERCC1 (rs3212986)	GG	271	94.22 ± 9.29	**<0.01**
	GT	195	53.47 ± 10.95	
	TT	62	136.74 ± 19.41	
***Urinary MDA***				
***Gene name (rs number)***	***Genotype***	***n***	***MDA (μM)***	***p***
ALDH2 (rs671)	AA	12	2.55 ± 0.39	**<0.01**
	AG	145	2.91 ± 0.11	
	GG	379	2.47 ± 0.07	
VAV3 (rs1410403)	AA	225	2.66 ± 0.09	**<0.01**
	AG	177	2.40 ± 0.10	
	GG	75	3.02 ± 0.16	
MGMT (rs12917)	CC	70	2.68 ± 0.14	**<0.01**
	CT	16	1.60 ± 0.30	
	TT	7	2.17 ± 0.45	
CYP3A4 (rs2242480)	CC	279	2.45 ± 0.08	**<0.01**
	CT	144	2.52 ± 0.11	
	TT	57	3.12 ± 0.18	
ADH1B (rs1229984)	AA	67	2.15 ± 0.17	**0.01**
	AG	111	2.67 ± 0.13	
	GG	26	3.01 ± 0.27	
XRCC3 (rs861539)	CC	55	2.47 ± 0.19	**0.02**
	CT	350	2.71 ± 0.07	
	TT	105	2.29 ± 0.14	
MPO (rs2333227)	CC	5	3.36 ± 0.62	**0.02**
	CT	283	2.42 ± 0.08	
	TT	98	2.82 ± 0.14	
DEFA4 (rs10103091)	AA	428	2.55 ± 0.07	**0.02**
	AT	95	2.86 ± 0.14	
	TT	7	1.59 ± 0.52	
DRD2 (rs1800497)	CC	223	2.76 ± 0.09	**0.04**
	CT	196	2.43 ± 0.10	
	TT	69	2.54 ± 0.17	
TP53 (rs1295105)	AA	277	2.62 ± 0.08	**0.05**
	AC	225	2.48 ± 0.09	
	CC	34	3.10 ± 0.24	

* Kruskal-Wallis tests.

## DISCUSSION

Our molecular epidemiological approach with in-depth biological monitoring can provide reliable mechanisms and health end points of tobacco smoking as an EDC, in our highly susceptible population. For an example of biomonitoring, tobacco smoking and the levels of cotinine showed association with a pronounced (about 50%) reduction in fecundability, resulting in a longer time-to-pregnancy^26^, because their developmental processes amplify or magnify the toxic responses via epigenetic or cell memory^27^. From the self-reported cigarette pack-years, the present adolescents consumed over 1/6 the amount of that of adults. However, urinary levels of cotinine in youth were 1/3 those of adults and urinary MDA or TTMA of youth were similar to the adults. Thus, youth can have high susceptibility to bio-produced tobacco metabolites with small amounts of tobacco.

Although cotinine and TTMA are metabolites of tobacco products, MDA is an exposure and response biomarker for tobacco smoking via oxidative stress from tobacco chemicals or their combustion. If we assume that youth’s answers for tobacco consumption were not biased due to self-report, we can note that youth can be highly susceptible to the bio-produced tobacco metabolites with small amounts of tobacco, compared to the adults. Around the onset of puberty, the activities of most of metabolic enzymes begin a gradual decline that continues throughout adolescence and concludes with attainment of adult capacity at the completion of pubertal development^28^.

In addition, a recent human liver study showed that the activities of CYP2A6 and CYP2E1, major metabolic enzymes of nicotine and benzene, were somewhat lower in adults (aged ≤69 years) than young people (21–45 years)^29^. Thus, the same exposure to tobacco smoking can result in high levels of metabolites of tobacco components among adolescents than adults and they can be more bioactive or toxic than the parent chemicals in tobacco.

Particularly, oxidative stress and aging can be a main mechanism and a health endpoint of tobacco smoking, respectively^30^. Chronic inhalation of cigarette smoke is a prominent cause of chronic obstructive pulmonary disease (COPD) and provides an important source of exogenous oxidants^31^. In the present study, we found urinary MDA, a biomarker for oxidative stress or reactive oxygen species (ROS), was associated with tobacco smoking as urinary cotinine. In addition, the growth rate of youth was negatively related to urinary MDA levels. To confirm the quality of the self-report, we recalled the questionnaire and confirmed the homogeneity of the answer for the growth rate with height and body weight. Thus, the present biological monitoring suggests tobacco reduces growth rate via oxidative stress in youth. As excessive ROS might react with nucleic acid, lipids, carbohydrates, and protein causing inflammation and oxidative stress that are the main causes for the development of various metabolic disorders^6^. Mitochondrial genome (mtDNA) instability contributes to mitochondrial dysfunction, and mtDNA mutagenesis may contribute to aging^32^. In addition, the exposure to ROS from tobacco smoking can cause a higher rate of mutations in the mitochondrial genome that accumulate over time and reduce the efficiency of mtDNA repair systems^33,34^. The present study also showed that youth smokers had somewhat high SNPs for inconsistent bases and significantly lower SNPs for consistent bases between buccal and blood cells than youth non-smokers or even adult smokers. Thus, high susceptibility in youth to tobacco smoking was confirmed with mtDNA alteration and MDA-related growth relay. The pathway analyses of exposure-related gene–gene interaction also suggest that MDA-related genes, MGMT, XRCC, and TP53, were known to interact for carcinogenesis, a degenerative disease. Moreover, a current pathway enrichment analysis showed some pathways for longevity and choline metabolism in cancer were associated with tobacco nicotine levels^35^.

Additional aging issues, EDCs including tobacco smoking can increase an overall risk of ovarian aging, leading to the diminish of ovarian reserve, decline of fertility or fecundity, irregularity of the menstrual cycle and an earlier age at menopause, and/or premature ovarian insufficiency/failure in epidemiological studies^8^. We also previously found tobacco smoking up-regulated aging genes, such as DEFA4 for hearing loss in adults^18^. Moreover, tobacco smoking-related oxidative stress has been emphasized in the appearance of the clinical manifestation of skin aging. Hexane-soluble tobacco smoke extract may induce matrix metalloproteinase-1 expression in human skin fibroblasts through the activation of the aryl hydrocarbon receptor pathway, which is pathogenetically involved in extrinsic skin aging. Thus, the present susceptibility/genetic biomarkers support that oxidative stress and aging are the mechanisms or health end points of tobacco smoking as an EDC.

### Strengths and limitations

As our study is based on in-depth biological monitoring, it has analytical strength and provides various evidence for tobacco smoking-related exposure and responses. In the present study, there are the time differences between collection and analyses of the samples, while its cross-sectional design cannot attribute causality. We also suggest that information on e-cigarette use should be included in future studies. In addition, mtDNA alterations are known to be related by aging and some of mtDNA alterations were related to smoking and age in this study. However, most of the mutation levels were quite lower than we expected. Thus, higher sensitive analyses for mtDNA alteration are needed from future studies.

## CONCLUSIONS

The present biological monitoring study assessed the impact of combustible cigarette smoking with various exposure, susceptibility and response biomarkers to clarify how tobacco smoking differently affects adolescents and adults in South Korea.

## Supplementary Material



## Data Availability

The data supporting this research can be found in the Supplementary file.
