# Supplementary material for: Tobacco smoking as an endocrine disrupting chemical: An assessment through biological monitoring
Source: Tob Induc Dis. 2025 Jul 29;23:10.18332/tid/205064. doi: 10.18332/tid/205064 (PMC12306452; doi:10.18332/tid/205064)
Supplement: Supplementary file 1 [file TID-23-109-s1.pdf]

Supplement table 1. List of the 96 selected SNPs and their frequencies in the subjects

Gene	rs NCBI	Nucleotide change	Amino-acid change	Minor allele frequency
**Tobacco smoking-response genes[1)]**				
ACTG1	rs1139405	C>T		0.22
	rs11657440	T>C		0.30
ARTN	rs2853224	A>C		0.43
	rs3848809	A>G		0.22
CXCR4	rs882300	A>G		0.28
DEFA1B	rs2979392	G>A		
DEFA4	rs10103091	T>A		0.18
	rs2738102	C>T		0.34
ELANE	rs3761007	C>T		0.09
FCGR3A	rs396991	T>G	Phe158Val	0.25
MARCH6	rs7720490	T>G		0.31
TGM2	rs4811528	G>A		0.31
	rs7270785	G>T		0.44
VAV3	rs1410403	G>A		0.26
	rs7528153	A>T	Thr298Ser	0.45
ACTG1	rs1139405	C>T		0.22
	rs11657440	T>C		0.30
ARTN	rs2853224	A>C		0.43
**Metabolism**				
ADH1B	rs1229984	A>G	His48Arg	0.21
ALDH2	rs671	G>A	Glu504Lys	0.06
CYP1A2	rs2472304	G>A		0.32
CYP1B1	rs1056836	C>G	Leu432Val	0.39
CYP2A13	rs8192789	C>T	Arg257Cys	0.07
CYP2A6	rs1137115	A>G		0.21
	rs11878604	T>C		0.22
	rs28399433	T>G		0.13
CYP2B6	rs3745274	G>T	Gln172His	0.27
CYP2C19	rs12777823	G>A		0.22

CYP2D6	rs35028622	C>A		0.40
CYP2E1	rs1329149	T>C		0.27
	rs3813867	G>C		0.10
CYP3A4	rs2242480	C>T		0.33
EPHX1	rs1051740	T>C	Tyr113His	0.32
	rs2234922	A>G	His139Arg	0.19
FMO3	rs1736557	G>A	Val257Met	0.09
	rs2266782	G>A	Glu158Lys	0.35
GSTM2	rs638820	T>C		0.43
	rs655315	T>C		0.48
GSTP1	rs1695	A>G	Ile105Val	0.33
NQO1	rs1800566	C>T	Pro187Ser	0.28
MPO	rs2333227	C>T		0.23
SULT1A1	rs9282861	G>A	Arg213His	0.23
UGT1A4	rs2011425	T>G	Leu48Val	0.11
UGT1A6	rs2070959	A>G	Thr181Ala	0.27
UGT2B15	rs1902023	T>G	Tyr85Asp	0.44
UGT2B7	rs7439366	T>C	Tyr268His	0.47
**Tobacco addiction**				
5-HT2A	rs6313	C>T		0.43
CHRNA3	rs12914385	C>T		0.31
	rs578776	C>T		0.46
	rs6495308	T>C		0.44
	rs6495309	C>T		0.34
CHRNB4	rs11072768	T>G		0.50
COMT	rs4680	G>A	Val158Met	0.39
	rs174699	C>T		0.16
DBH	rs5320	G>A	Ala211Thr	0.11
DRD2	rs1800497	C>T	Glu713Lys	0.30
HTR3A	rs10160548	G>T		0.45
LAMB4/NRCAM	rs382140	A>G		0.25
NCALD	rs16868941	G>A		0.14
NET-8	rs5569	C>T		0.27
OPRM1	rs1799971	A>G	Asn40Asp	0.19
SLC6A3	rs27072	C>T		0.19

SLC6A4	rs2020936	C>T		0.23
TPH1	rs1799913	C>A		0.36
	rs1800532	C>A		0.36
TPH2	rs4570625	G>T		0.36
**Tobacco smoke induced diseases**				
AHR	rs2066853	G>A	Arg554Lys	0.27
AHRR	rs2292596	C>G	Pro189Ala	0.31
ALOX5	rs4948671	C>T		0.25
	rs7099684	A>T		0.16
AOX1	rs17593621	T>C		0.23
	rs12621063	C>A		0.29
ARNT	rs12410394	G>A		0.39
CSK	rs1378942	G>T		0.33
DNER	rs7594321	T>C		0.23
HPRT1	rs6634990	T>G		0.45
IKBKE	rs10863430	T>G		0.14
IL17A	rs4711998	A>G		0.46
KCNJ2/SOX9	rs11654749	G>T		0.34
PPARG	rs3856806	C>T		0.12
TP53	rs1042522	C>G	Pro72Arg	0.40
	rs12951053	A>C		0.16
WRN	rs1801195	G>T	Leu1074Phe	0.47
**Epigenetic modulation**				
HDAC1	rs1741981	T>C		0.49
MTHFR	rs1801133	C>T	Ala222Val	0.32
**DNA repair**				
ERCC1	rs4253211	G>C	Arg1230Pro	0.06
	rs11615	T>C		0.36
	rs2298881	C>A		0.19
	rs3212961	C>A		0.26
	rs3212986	G>T	Gln504Lys	0.29
MGMT	rs12917	C>T	Leu84Phe	0.15
MSH3	rs1042821	C>T	Gly39Glu	0.19

	rs26279	G>A	Ala1045Thr	0.31
OGG1	rs1052133	C>G	Ser326Cys	0.30
XPC	rs2228001	C>A	Lys939Gln	0.34
	rs2228000	C>T	Ala462Val	0.25
XRCC1	rs1799782	C>T	Arg194Trp	0.13
	rs25487	A>G	Gln399Arg	0.26
XRCC3	rs861539	C>T	Thr241Met	0.25

1) based on Na et al. 2015

Supplement table 2. Alteration of mt DNA by smoking and gender

Item *	Group	mean	stdev	P value
Deletion of bases inconsistent between buccal and blood cells	M-nonsmoker	.01	.10	.17
	F-nonsmoker	.00	.00	
	M-smoker	.00	.00	
Deletion of bases consistent between buccal and blood cells	M-nonsmoker	.09	.29	.66
	F-nonsmoker	.11	.32	
	M-smoker	.08	.28	
Deletion of bases in blood cells	M-nonsmoker	.00	.07	.41
	F-nonsmoker	.00	.00	
	M-smoker	.00	.00	
Deletion of bases in oral(buccal) cells	M-nonsmoker	.01	.07	.41
	F-nonsmoker	.00	.00	
	M-smoker	.00	.00	
SNPs for inconsistent bases between buccal and blood cells	M-nonsmoker	.47	1.27	.46
	F-nonsmoker	.32	1.03	
	M-smoker	.47	1.77	
SNPs for consistent bases between buccal and blood cells	M-nonsmoker	1.16	1.12	.13
	F-nonsmoker	1.34	1.32	
	M-smoker	1.02	1.05	
SNPs in blood cells	M-nonsmoker	.22	.77	.78
	F-nonsmoker	.16	.53	
	M-smoker	.18	.72	

SNPs of bases in oral(buccal) cells	M-nonsmoker	.25	.89	.49
	F-nonsmoker	.16	.75	
	M-smoker	.29	1.58	

Note: M-nonsmoker, N=192; F-nonsmoker, N=187; M-smoker, N=156; total N=534 (Due to poor DNA quality, 86 samples could not be analyzed for mt DNA mutation.)

*deletion of bases or substitution of bases (SNP) between 100-322 bp of HV2 region

© 2025 Lee H.S. et al.
